# ToxDB: pathway-level interpretation of drug-treatment data

**DOI:** 10.1093/database/baw052

**Published:** 2016-04-13

**Authors:** C. Hardt, M.E. Beber, A. Rasche, A. Kamburov, D.G. Hebels, J.C. Kleinjans, R. Herwig

**Affiliations:** 1 Department of Computational Molecular Biology, Max-Planck-Institute for Molecular Genetics, Ihnestr, 73, D-14195 Berlin, Germany; 2 Department of Toxicogenomics, School of Oncology and Developmental Biology (GROW), Maastricht University, Maastricht, Md 6200, The Netherlands; 3 Department of Cell Biology-Inspired Tissue Engineering, MERLN Institute, Maastricht University, Universiteitssingel 40, Maastricht, Er 6229, The Netherlands

## Abstract

**Motivation:** Extensive drug treatment gene expression data have been generated in order to identify biomarkers that are predictive for toxicity or to classify compounds. However, such patterns are often highly variable across compounds and lack robustness. We and others have previously shown that supervised expression patterns based on pathway concepts rather than unsupervised patterns are more robust and can be used to assess toxicity for entire classes of drugs more reliably.

**Results:** We have developed a database, ToxDB, for the analysis of the functional consequences of drug treatment at the pathway level. We have collected 2694 pathway concepts and computed numerical response scores of these pathways for 437 drugs and chemicals and 7464 different experimental conditions. ToxDB provides functionalities for exploring these pathway responses by offering tools for visualization and differential analysis allowing for comparisons of different treatment parameters and for linking this data with toxicity annotation and chemical information.

**Database URL:** http://toxdb.molgen.mpg.de

## Introduction

The assessment of toxicity of compounds such as drugs, industrial chemicals, cosmetics and food ingredients is an important aspect of research with implications for patient health, consumer protection and nutrition.

In order to identify more reliable molecular predictors of toxicity huge amounts of toxicogenomics data have been generated worldwide, e.g. by the Japanese Toxicogenomics project (1), the US Drug Matrix project (2) and the European carcinoGENOMICS project (3). By far the largest part of toxicogenomics data targets the transcriptome and is generated with microarrays. The goals of these projects are to identify gene sets that are predictive of cellular toxicity, to classify the toxic hazard, and to quantify the toxic risk of the compounds. However, the discriminatory potential of gene expression patterns is limited and lacks robustness across studies (4). Thus, we (5) and others (6) have shown previously that the predictive power of gene expression data could be improved when incorporating molecular networks, in particular, pathway concepts.

In this work, we take advantage of the pathway collection of ConsensusPathDB (7), a meta-database of human molecular interactions that integrates the content of 12 publicly accessible pathway databases with a total of 4593 human pathway concepts. Furthermore, we have previously published a method for quantifying pathway responses from gene expression data (5), and in this study we used this method in order to provide pathway-level response data for 437 chemical compounds across several different experimental conditions. We have built a database, ToxDB, which provides functionalities for visualization and differential pathway analysis along with toxicity and chemical annotation which gives researchers the possibility to better characterize the functional consequences of drug exposure.

## Toxdb workflow

**Figure 1.**
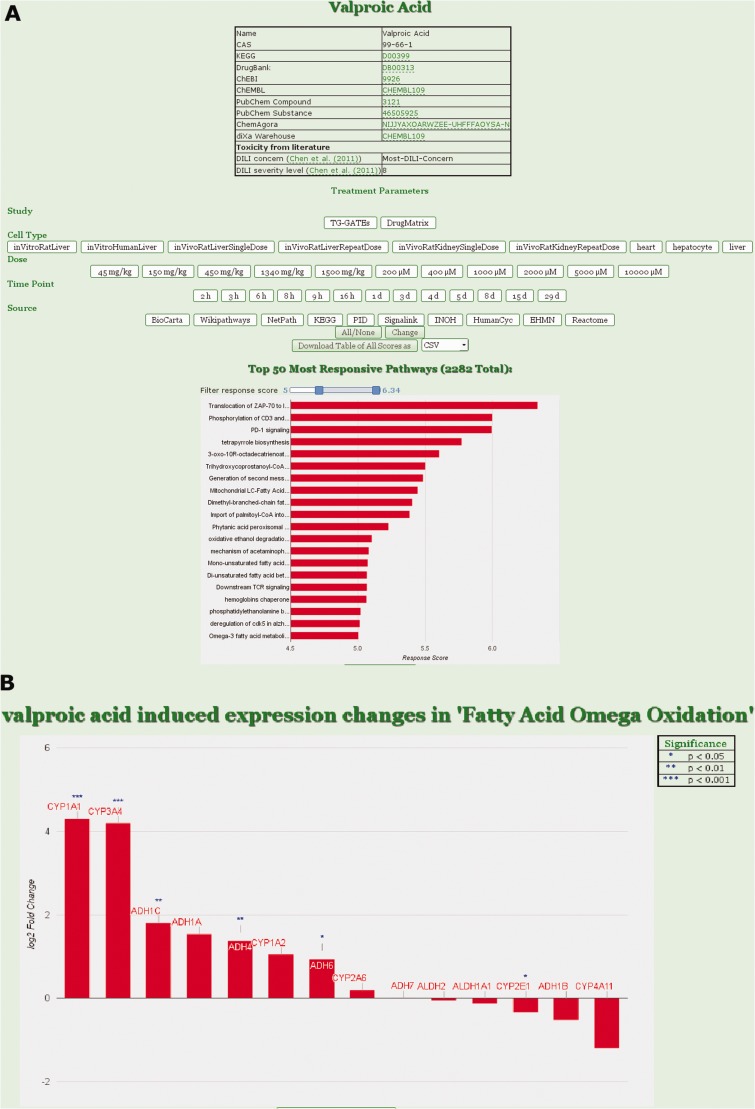
ToxDB web interface. **(A)** Drug view in ToxDB. Treatment parameters can be set and the responding pathways are shown with a bar plot in decreasing order. Number of pathways visualized can be set by the user according to RPR score with a slider; chemical information for the compound is interlinked. **(B)** Gene view in ToxDB. For each pathway the corresponding genes associated with that pathway can be visualized. The statistical results derived from the series of replicated experiments are displayed in the table on top of the graph (not shown here).

ToxDB builds on three components: (i) a comprehensive collection of pathway concepts along with drug treatment microarray data, (ii) a numerical method to compute pathway responses from genome-scale expression data, (iii) a web interface that enables user interaction (Figure 1).

### Gene expression data and molecular pathways

ToxDB is currently based on gene expression data from two large-scale studies comprising a total of 7464 different experiments (437 different chemical compounds) in human and rat tissues at different time points and with different drug dosages. The first study (Open TG-GATES) provides toxicity information on compounds tested in rat *in vivo*, liver and kidney cells, and in human hepatocytes (8). The second study (DrugMatrix) provides toxicogenomic profiles of compounds tested in rat liver, kidney, heart and muscle tissues (2). The 4593 molecular pathway concepts are derived from the ConsensusPathDB, release 31 (7). In order to increase the robustness of pathway response and exclude smaller pathways (e.g. simple reactions), selection of pathways was restricted to those 2694 that had ≥5 gene members with measured expression values.

### Microarray data pre-processing

We used custom cdf files for mapping oligonucleotide probes of the human and rat microarrays to respective genes (9). This results in a unique assignment of a probe to a gene locus and in a varying number of probes per gene (≥3 probes per gene).

Replicate experiments for a treatment with a certain dosage and at a certain time-point along with the corresponding control replicate experiments were grouped and the raw data was normalized using the GC Robust Multiarray Average method.

### Orthology mapping

In the case of human data, genes could directly be related to human pathways. In the case of rat data, genes were assigned to human pathways by orthology. We used the orthology mapping of the Ensembl Biomart repository (10). We limited the mapping to ‘one2one’ and ‘one2many’ homology relationships:

- One2one: the rat gene has exactly one orthologous human gene, and the corresponding rat microarray value is assigned to that human gene.
- One2many: the rat gene has multiple orthologs in the human genome, and the corresponding rat microarray value is assigned to all human paralogs.

### Pathway scoring

The pathway scoring method was previously developed by us in the context of discriminating classes of chemicals with respect to their carcinogenic hazard in stem cell-derived human hepatocytes (5). We define a relative pathway response (RPR) scoring method which computes for each pathway a numerical value that quantifies its response measured with gene expression microarrays (or alternatively RNA-sequencing). Although we predominantly work with gene expression data, in principle there is no restriction to this type of data and experimental values could be any quantitative read-out from transcriptomics or proteomics experiments.

A pathway path*_k_* is defined as a set of genes $M_{k}=\left\{g_{1},\ldots ,g_{n}\right\}$ of size $\left|M_{k}\right|=n_{k}$. ToxDB uses the ConsensusPathDB (htttp://consensuspathdb.org) as the pathway resource since it summarizes the major publicly available pathway databases.

Suppose genome-wide case-control experiments are carried out with some material, e.g. human tissue, rat tissue, cell lines including replicates. In toxicology such experiments consist typically of several chemical treatments of the cells which are compared against the untreated control cells at matched time points. We apply the pre-processing described earlier and compute a statistical test for all case-control studies. The choice of the statistical test is dependent on the type of data and the corresponding model for the background distribution. Suitable test-procedures for microarray data could be, e.g. limma, Student’s *t*-test, Welch test or Wilcoxon’s rank sum test, for RNA-sequencing data it could be, for example DEXSeq and edgeR. In this study, we performed Student’s *t*-test for each case-control experiment.

This yields for each gene $g_{i}$ and each chemical *j*, a fold-change $r_{ij}$ (computed as the ratio of the mean expression values of treatment and control replicates) and a *P*-value $p_{ij}$ (judging the significance of the fold-change given the null hypothesis of no change of expression).

We now compute a gene score $s_{ij}$ for each gene $g_{i}$ and each chemical *j* by:

$$s_{ij}={|\log}_{2}r_{ij}||\log_{10}p_{ij}|$$

The gene score describes a weighted fold-change of the gene with respect to the particular treatment, whereby the weight is increasing with the significance of the fold-change. Although technically, Student’s *t*-test procedure can be computed even with very low sample sizes, it is clear that the test has higher power the more replicates are used. However, in most toxicogenomics studies sample sizes are rather low, in the range of two to five replicates, which may downsize the confidence of the significance computation. On the other hand, using the above procedure incorporates all expression data and avoids a statistical pre-selection of genes based on *P*-values. Instead, *P*-values are only used for additional weighting of expression fold-changes which seems a more appropriate approach in these cases.

Furthermore, it should be noted that gene scores do not distinguish between positive or negative gene expression regulation but rather reflect whether the gene is affected by the treatment or not.

The pathway score path*_kj_* for pathway path*_k_* and chemical *j* is defined as the average gene score of all genes assigned to the pathway:

$${path}_{kj}=\frac{1}{n_{k}}\sum_{g_{i}\in M_{k}}s_{ij}.$$

In order to make pathway scores comparable across different treatments, we divide each score by the median pathway score over all pathways, path*_ij_*, and compute the relative response score (RPR):

$${RPR}_{kj}=\log_{2}\left(\frac{{path}_{kj}}{median\left({path}_{ij}|i\right)}\right).$$

**Figure 2.**
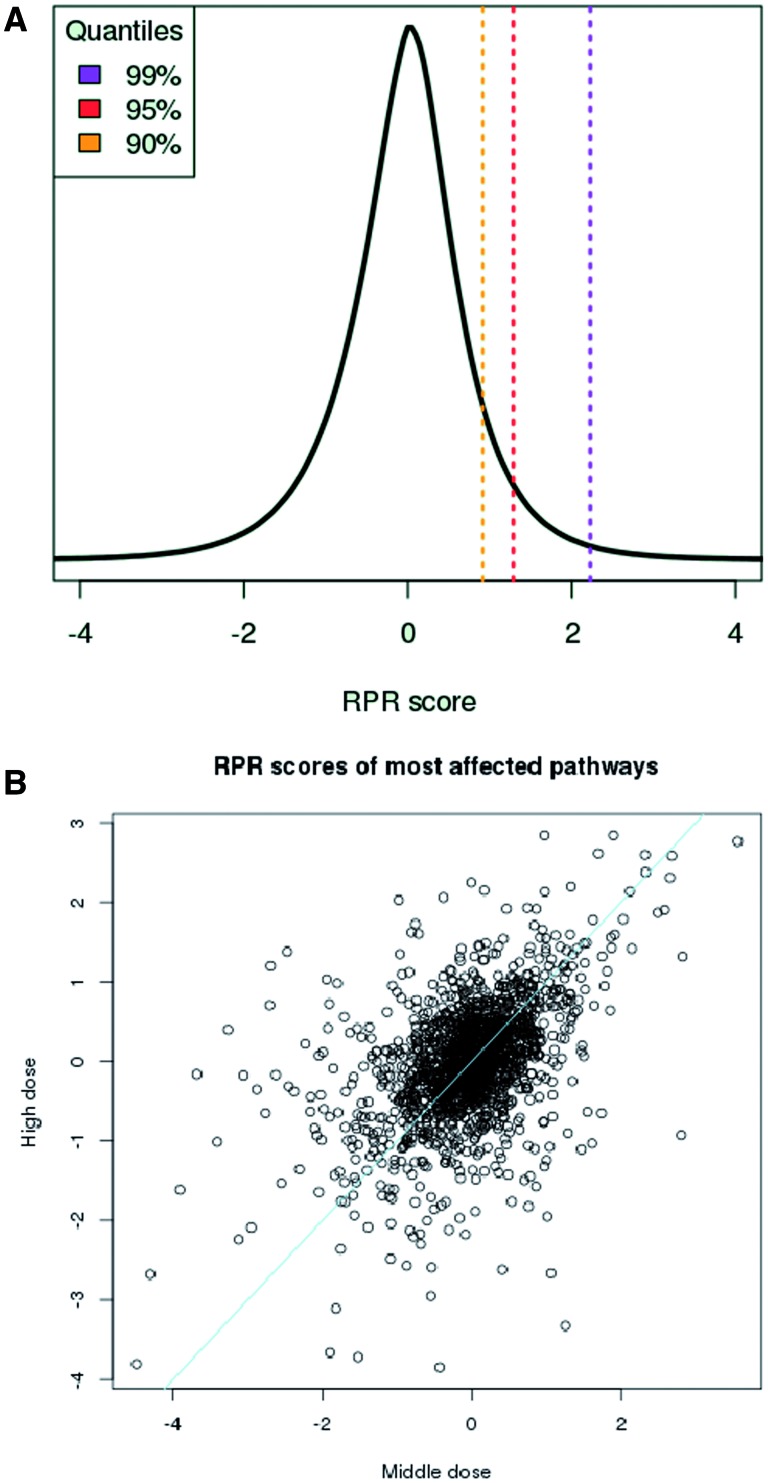
Measuring pathway response. **(A)** The RPR scores are Gaussian-distributed and comparable across different compound treatment experiments. **(B)** Pathway scores, path_kj_, reflect chemical dose. Scores derived from ‘middle’ (X-axis) and ‘high’ (Y-axis) doses for responding pathways across 64 different treatments increase with dosage. Drugs were classified by Chen *et al.* (11) as having ‘less’ and ‘most’ concern, respectively for drug-induced liver injury and gene expression data was taken from TG-GATES human in vitro hepatocyte data. Line, equal response.

The consequence of the transformation is that in all treatments half of the pathways get negative RPRs and half of them get positive ones. RPRs are comparable across different treatments and follow a Gaussian distribution (Figure 2A). Thus, higher RPRs reflect significant pathway responses to the chemical treatment. Furthermore, pathway scores, path*_kj_*, reflect the strength of chemical dose (see Figure 2B) which is a necessary condition when quantifying pathway responses.

It should be noted that the pathway scores are fairly robust across different subsets of data and, thus, that the distribution of all RPR scores can serve as a background distribution for judging significance of individual RPR scores (Supplementary Materials). In the web interface the user is thus provided with the background distribution derived from the entirety of RPR scores when inspecting individual responses.

### Web interface

The backend of the ToxDB is composed of a PostgreSQL database (version 9.2.4) running on an Apache/2.4.4 (64-bit Unix) server. The frontend HTML is designed using Flask (version 0.10.1), a web framework for Python (version 3.4.1). Pathway data are currently based on release 31 of the ConsensusPathDB and will be updated regularly. Plots in the web interface are drawn using JavaScript and the Google Visualization tools.

The ToxDB web interface provides functionalities that allow exploration of different aspects of drug treatment data.

#### Search function

On the front page, users can do a comprehensive search of all drugs, pathways, and genes in the database. Singular words, logical operators as well as several specific IDs are accepted.

#### Compare

Here, treatments can be compared by assigning them to groups and the differences of the two groups are quantified by Student’s *t*-test. This feature can be used for example when inferring pathways that are affected differentially by different sets of drugs, in different target organs or different species.

#### Browse drugs

Users can select from the list of available drugs. The resulting view shows chemical annotation of this compound as well as a menu where experimental conditions can be fixed, e.g. study, cell type, dosage and time point. After setting the conditions, the corresponding expression data are assessed and the resulting pathway concepts are shown ordered by their RPR scores. A slider can be used to specify the number of displayed pathways. By clicking on a specific pathway, users can switch from the pathway (Figure 1A) to the gene view (Figure 1B) displaying all genes that are annotated for the pathway and for which expression data are available. Gene fold-changes from the experiment (treatment vs normal) are displayed as bar plots.

#### Browse pathway

Conversely, if the user is interested in a certain pathway, e.g. when evaluating a specific functional assay, a single pathway can be selected and the resulting response scores of this pathway are displayed across all compounds and treatments. Additionally, toxicity information from two independent reviews (11, 12) is overlaid.

#### Download

Tables and plots resulting from the described tools are made available for download in various formats in order to use the data in further analyses. Additionally, we provide a download section for the use cases reported in this study (Supplementary Materials).

#### External links and additional information

Compound information is made available along with external links to several other resources:

Chemical Abstracts Service registry number

Drug information according to several databases (KEGG, DrugBank, ChEMBL, PubChem compound and substrate and the ChemAgora meta-database).

diXa warehouse for chemical safety information and for experimental data download (13).

Toxicity information—for judging liver toxicity of the compounds we included two assignment procedures: the first was defined by the FDA using box readings and literature mining (11) and the second uses structure–affinity relationships (12).

### Future updates

ToxDB will be updated on a regular basis, approximately every six months. Updates will include pathway concepts from the ConsensusPathDB (7) as well as gene ID and orthology mappings from the Ensembl database (10). Currently, ToxDB features expression data from two studies, Open TG-GATEs (1) and DrugMatrix (2), but additional sources like carcinoGENOMICS (3) and Connectivity Map (14) are planned to be incorporated. Furthermore, additional statistical tests for pre-processing the microarray data are planned that take into account not only individual case-control studies but rather the full time series and dosage administrations. Additionally, a future update of the web interface will include the possibility of uploading user-defined pathway signatures that could then be screened for similarity across the body of characterized compounds with a connectivity map-based approach (14).

## Application

In order to exemplify work with ToxDB we have conducted several use cases, in which we investigate the effects of different drug treatments on cellular pathway responses.

In the first use case we investigated five drugs with known hepatotoxic or cardiotoxic effects, respectively. By investigating drug-induced gene and pathway responses in ToxDB we can confirm information on toxicity and modes of action derived from literature and other databases, like ChEMBL. These results encourage inspecting other, less well-studied drugs in ToxDB and their effects on genes and pathways.

In the second use case we approach the problem of identification potential modes of action in the opposite way. Here, we start from certain disease-related pathways and infer the drugs that mostly affect the pathway. We focus on the ‘cytochrome P450 pathway’ and on ‘non-alcohilc fatty liver disease’, which both play a role in hepatotoxicity. We find that they are indeed mostly affected by drugs known to promote liver disease, e.g. rifampin (11). Although this serves as a confirmation of previous studies, the same approach can be used to identify other drugs that have not been shown to be hepatotoxic. Similarly, one can look at completely different pathways related to other diseases (e.g. cancer).

Full details of the use cases are reported in the Supplementary Materials.

## Conclusion

ToxDB is a resource that analyzes drug-induced gene expression changes at the pathway level. Although the current approach is focused on drug treatment data, the pathway analysis approach can be applied to more general scenarios where case-control studies are given (e.g. disease vs control states, tumor vs normal). By elevating analyses from the gene to the pathway level it is possible to gain more general information on functional changes and more robust biomarkers what ultimately contributes to the improvement of drug development pipelines.

## Supplementary data

Supplementary data are available at Database Online.
